# Epigenetic dysregulation in pancreatic cancer: emerging biomarkers and clinical applications

**DOI:** 10.3389/freae.2025.1723159

**Published:** 2025-11-27

**Authors:** Hina Sultana, Abhishikt David Solomon, Atif Khurshid Wani, Surendra K. Shukla

**Affiliations:** 1 Integrative Program in Biological and Genome Sciences, University of North Carolina at Chapel Hill, Chapel Hill, NC, United States; 2 Division of Oral and Craniofacial Health Sciences, Adams School of Dentistry, University of North Carolina at Chapel Hill, Chapel Hill, NC, United States; 3 Department of Biotechnology, School of Bioengineering and Biosciences, Lovely Professional University, Jalandhar, Punjab, India; 4 Department of Oncology Science, University of Oklahoma Health Sciences Center, Oklahoma City, OK, United States

**Keywords:** pancreatic ductal adenocarcinoma (PDAC), DNA methylation biomarkers, liquid biopsy, epigenetic therapies, clinical translation, non-coding RNAs, miRNA biomarkers, lncRNA biomarkers

## Abstract

Pancreatic cancer, in particular pancreatic ductal adenocarcinoma (PDAC), is one of the most lethal malignancies with delayed diagnosis, aggressive nature, and profound therapeutic resistance. Recent research has emphasized epigenetic dysregulation not as a bystander but as a driving force in pancreatic tumorigenesis, progression, metastasis, and immune evasion. This review presents a detailed analysis of the multifaceted functions of epigenetic mechanisms like DNA methylation, histone modifications, and non-coding RNAs in shaping the pancreatic cancer epigenome. We discuss how these alterations fuel tumor heterogeneity, modulate the tumor microenvironment, and interact with key oncogenic mutations like *KRAS* and *TP53*. Further, we discuss the potential of epigenetic alterations as diagnostic, prognostic, and predictive biomarkers with an emphasis on their application in liquid biopsies. Finally, we evaluate current and prospective epigenetic therapies, their cooperation with chemotherapy and immunotherapy, and the prospects of new approaches such as CRISPR-based epigenome editing and personalized epigenetic profiling. Together, these findings point to the epigenome as a powerful window of opportunity for understanding, diagnosing, and eventually targeting pancreatic cancer.

## 1 Introduction

Pancreatic cancer, especially PDAC, is one of the biggest challenges of modern oncology. In the US alone, 67,440 people will be diagnosed with pancreatic cancer in 2025, and approximately 51,980 will die from the disease. It is the third most common cause of cancer-related death in the US, and data show that it is predicted to become the second most common cause of cancer-related death within the next several years (Facts About Pancreatic Cancer, 2025). Pancreatic cancer was the 12th most common cause of cancer in 2022 in the world with 510,992 new cases, but the sixth most common cause of cancer-related death in the world in 2022, responsible for 467,409 deaths, and also the sixth most common cause of cancer-related death in 2021 (Pancreatic cancer statistics, 2022; Bray et al., 2024). Its characteristically late diagnosis, aggressive biological behavior, and extreme resistance to conventional therapies are the causes of a very poor prognosis. The US relative survival at 5 years for all combined stages is approximately 13%. If diagnosed with the most common form, pancreatic adenocarcinoma, survival at 5 years is even poorer, at 8%. For advanced stages of cancer, a 5-year survival of as little as 2–9% has been documented (Sesock, 2025). Such clinical necessity and heavy socioeconomic burden have stimulated strong research into the molecular underpinnings of the disease. Aside from the genetically well-characterized mutations as cancer architects, the science of epigenetics, inheritance of gene expression modifications that are not reliant on changes to the underlying DNA sequence, has emerged as a crucial, dynamic level of regulation profoundly implicated in pancreatic carcinogenesis.

The reversibility of epigenetic modifications is an intrinsic property that holds an interesting potential for therapeutic targeting, in contrast to the relatively irreversible character of genetic mutations. This plasticity, the ability to be reshaped, is precisely the reason why epigenetic treatments are the focus of earnest inquiry. But the same plasticity, making the epigenetic marks available for targeting, is one that can be exploited by cancer cells. Pancreatic cancer is exceedingly well recognized to have the capability of becoming resistant to various therapies. The plasticity of the epigenome can allow cells to reprogram gene expression for adaptation to environmental stress, such as the deluge of therapeutic agents. Therefore, the same reversibility that leads to a therapeutic window can be hijacked by the cancer cells to epigenetically reprogram and get around epigenetic therapy or treatments once more. This means that epigenome-based therapies will have to be complex, perhaps using combination treatments or adaptive treatment schedules in order to outsmart this cell versatility.

This review will outline the varied functions of epigenetic processes in pancreatic cancer, including how their disruption fuels tumorigenesis, structures the tumor microenvironment, offers new promise as biomarkers, and presents hopeful, if daunting, targets for treatment. Unraveling these enigmatic epigenetic choreographies is not a matter for the ivory tower; it promises actual revelations of new pathways to improved therapies for a disease where advances have been painfully slow.

## 2 Overview of pancreatic cancer

### 2.1 Defining pancreatic cancer and its clinical impact

Pancreatic cancer originates from cells within the pancreas, an organ nestled behind the stomach that serves dual vital roles: producing enzymes essential for digestion (its exocrine function) and manufacturing hormones like insulin and glucagon to regulate blood sugar levels (its endocrine function) (Chrystoja et al., 2013). The disease is fundamentally characterized by the uncontrolled proliferation of these cells, which amass to form tumors. These tumors possess the ominous ability to invade adjacent tissues and, frequently, to metastasize, spreading to distant organs such as the liver and lungs. Pancreatic cancer is notorious for its aggressive biological behavior and consequently, its grim prognosis (Bao et al., 2010; Halbrook et al., 2023). A significant challenge is that it often remains asymptomatic in its early, more treatable stages, leading to diagnosis when the disease is already advanced and curative options are severely limited. Globally, it ranks as a leading cause of cancer-related mortality, underscoring the urgent need for improved diagnostic and therapeutic strategies.

### 2.2 Tumor subtypes

The term “pancreatic cancer” encompasses several distinct pathologic entities. The most common, accounting for approximately 95% of all cases, is PDAC (Park et al., 2021). PDAC arises from the epithelial cells lining the ducts of the exocrine pancreas and is the most common and lethal form (Feig et al., 2012), thus forming the subject of this review and most research studies.

Less common are pancreatic neuroendocrine tumors (PNETs), which develop from the pancreas hormone-secreting endocrine cells (Halfdanarson et al., 2008). PNETs often exhibit dissimilar biologic behaviors, possess varied risk factors, are differently diagnosed, and respond to alternate therapies than PDAC. Adenosquamous carcinomas, squamous cell carcinomas, and acinar cell carcinomas are additional unusual exocrine subtypes with varying characteristics (Luo et al., 2019).

Also, not all growths in the pancreas are cancerous. Some are benign, e.g., serous cystic neoplasms (SCNs), while others are pre-cancerous lesions, e.g., mucinous cystic neoplasms (MCPNs) and intraductal papillary mucinous neoplasms (IPMNs) (Basturk et al., 2009). These pre-cancerous lesions, particularly some IPMNs and MCPNs, may progress to invasive cancer if not treated or managed appropriately. Progression from normal pancreatic tissue to invasive PDAC through precursor lesions such as Pancreatic Intraepithelial Neoplasia (PanIN) is an accepted model of pancreatic carcinogenesis (Maitra et al., 2003; Hruban et al., 2008).

While genetic mutations are established causes of pancreatic cancer, the profound biological and clinical distinction between subtypes like PDAC and PNETs, and the critical transformation from pre-cancerous lesions to invasive carcinoma, will also involve substantial and subtype-specific epigenetic reprogramming. The progression from a pre-malignant lesion, such as an IPMN or PanIN lesion, to an invasive PDAC is a multi-step process in which epigenetic alterations are not merely ancillary changes but rather early and driving events. These epigenetic alterations, which precede or coincide with genetic mutations, can orchestrate the changes in cell identity and cell behavior that define malignancy. This observation offers the possibility of screening for high-risk individuals, developing biomarkers for early detection at these precursor stages, or even developing interventions to prevent or reverse the development towards invasive cancer.

### 2.3 Tumor microenvironment and desmoplasia

One characteristic and progressively challenging aspect of PDAC is its unique and highly immunosuppressive tumor microenvironment (TME) (Lafaro and Melstrom, 2019; Hessmann et al., 2020). The TME is not merely a silent backdrop for growth of the tumor cells but an active participant in disease progression and treatment resistance. One hallmark of the PDAC TME is a strong fibrotic reaction named desmoplasia. This dense, scar tissue-like stroma may constitute more than 80% of total tumor mass and forms a formidable biological and physical barrier (Young et al., 2018; Looi et al., 2019).

Desmoplastic stroma is an advanced ecosystem of the abundance of extracellular matrix (ECM) proteins, such as several types of collagen, fibronectin, and hyaluronic acid. It comprises a number of cell populations, the most distinguishing of which are activated pancreatic stellate cells (PSCs), which are differentiated into myofibroblastic cancer-associated fibroblasts (CAFs) (Pandol et al., 2009; Wilson et al., 2014; Schnittert et al., 2019; Bulle and Lim, 2020). Immune cells like immunosuppressive cells such as regulatory T cells and myeloid-derived suppressor cells also infiltrate this stroma, along with many cytokines, growth factors, and ECM-modifying enzymes (Mahadevan and Von Hoff, 2007; Tjomsland et al., 2011; Sperb et al., 2020).

The desmoplastic stroma has multiple detrimental functions. It has a function of actively nourishing tumor development, facilitating invasion locally, and assisting metastatic spread. Of paramount importance, it acts as a physical barrier that interferes with drug delivery and function of therapeutic agents, both chemotherapeutic and immunotherapy, by creating interstitial fluid pressure and collapsing blood vessels, leading to a hypovascularity. Additionally, the cellular and molecular components of the desmoplastic stroma also conspire to create a profoundly immunosuppressive milieu, shielding tumor cells from immune attack and also playing a role in the notorious drug insensitivity of PDAC (Feig et al., 2012; Provenzano et al., 2012; 2012; Provenzano and Hingorani, 2013; Lunardi et al., 2014; Martinez-Bosch et al., 2018; Hosein et al., 2020).

The dynamic and complex interaction between cancer cells and stroma is not a one-way process but active bidirectional communication. This crosstalk is now known to be orchestrated, at least partially, by epigenetic processes in both the cancer cells and the numerous populations of stromal cells. Cancer cells release a broad spectrum of signaling molecules; cytokines, growth factors, and extracellular vesicles, that can cause epigenetic reprogramming in nearby stromal cells such as PSCs and fibroblasts (Hwang et al., 2008; Dunér et al., 2011). This reprogramming is able to activate these stromal cells, compelling them to take on a pro-tumorigenic phenotype characterized by increased ECM deposition, release of growth factor-promoting factors, and development of an immunosuppressive niche (Hwang et al., 2008; Waghray et al., 2013). Activated stromal cells can, in turn, release epigenetically acting factors that impact the cancer cells, leading to proliferation, survival, invasiveness, and resistance to therapy. This concept is buttressed by evidence that inhibiting epigenetic regulators will inhibit tumor cells from activating these tumor-supportive phenotypic shifts in adjacent stromal cells, and thereby inhibit the formation of the characteristic dense stroma. Therefore, targeting the TME’s own epigenetic regulatory machinery, or disrupting the epigenetic mechanisms that govern cancer and stromal cell harmful communication, emerges as a pivotal and potentially amenable axis. Such strategies might attempt to dismantle this protective niche, normalize the TME, and thereby enhance the efficacy of conventional and innovative therapies.

## 3 Molecular basis of epigenetic regulation

Epigenetics is the term used to describe the molecular processes that control gene expression but do not change the underlying DNA code. These events are heritable during cell division and are of immense significance for normal development, cellular differentiation, and environmental adaptation (Berger et al., 2009). In cancer, though, this epigenetic regulation is disrupted, producing a cacophony of dysfunctional gene expression that drives malignant transformation and progression. The chief conductors of this epigenetic regulation are DNA methylation, histone modification, chromatin remodeling, and non-coding RNAs.

### 3.1 DNA methylation

DNA methylation is one of the most extensively studied epigenetic modifications. It involves the covalent addition of a methyl group (CH3) to the 5-carbon position of a cytosine pyrimidine ring, typically occurring within CpG dinucleotides; regions where a cytosine nucleotide is followed by a guanine nucleotide in the linear sequence. Clusters of CpG dinucleotides, known as CpG islands, are frequently found in the promoter regions of genes (Robertson and Jones, 2000; Strathdee and Brown, 2002; Shenker and Flanagan, 2012; Ambrosi et al., 2017; Gujar et al., 2019). The methylation process is catalyzed and maintained by a family of enzymes called DNA methyltransferases (DNMTs). DNMT1 is primarily responsible for copying pre-existing methylation patterns onto newly synthesized DNA strands during replication, thus ensuring the heritability of these marks (maintenance methylation). DNMT3A and DNMT3B are involved in establishing new methylation patterns (*de novo* methylation) during development or in response to cellular signals (Jones and Liang, 2009; Jurkowska et al., 2011; Chen and Chan, 2014; Ren et al., 2018; Gujar et al., 2019; Chen and Zhang, 2020).

Generally, when DNA methylation occurs at CpG islands within gene promoter regions, it is associated with stable transcriptional silencing (Deaton and Bird, 2011). This silencing can occur through several mechanisms: the methyl groups can physically hinder the binding of transcription factors and other components of the transcriptional machinery to the DNA, or they can recruit methyl-CpG-binding domain proteins (MBDs) which, in turn, enlist co-repressor complexes that promote a condensed, inaccessible chromatin state (heterochromatin) (Clouaire and Stancheva, 2008; Du et al., 2015). Aberrant DNA methylation patterns, characterized by both widespread (global) hypomethylation and gene-specific (local) hypermethylation, are a hallmark of many human cancers, including pancreatic cancer, where they contribute to genomic instability and the silencing of tumor suppressor genes (Bararia et al., 2020).

### 3.2 Histone modifications

Histones are highly conserved basic proteins that package and order DNA into structural units called nucleosomes, which are the fundamental building blocks of chromatin. Each nucleosome consists of an octamer of four core histone proteins (H2A, H2B, H3, and H4), around which approximately 147 base pairs of DNA is wrapped. The N-terminal tails of these histone proteins protrude from the nucleosome core and are subject to a remarkable variety of post-translational modifications (PTMs). These PTMs include acetylation, methylation, phosphorylation, ubiquitination, sumoylation, and others. The combinatorial patterns of these modifications on histone tails are often referred to as the “histone code”. This “code” is interpreted by other proteins, which then influence chromatin structure and dictate whether genes are active or silent (Strahl and Allis, 2000).

Histone acetylation, the addition of an acetyl group to lysine residues, is generally associated with transcriptional activation. This modification is catalyzed by enzymes called histone acetyltransferases (HATs). Acetylation neutralizes the positive charge of lysine residues, weakening their interaction with the negatively charged DNA backbone. This leads to a more relaxed, open chromatin structure (euchromatin), making the DNA more accessible to transcription factors and the transcriptional machinery. Conversely, the removal of acetyl groups by histone deacetylases (HDACs) restores the positive charge on lysines, promoting a more condensed chromatin structure and gene repression (Bannister and Kouzarides, 2011; Shvedunova and Akhtar, 2022).

Histone methylation involves the addition of methyl groups to lysine or arginine residues. Unlike acetylation, which is generally activating, histone methylation can be associated with either transcriptional activation or repression, depending on the specific amino acid residue modified (e.g., lysine 4 on histone H3, H3K4, versus lysine 27 on histone H3, H3K27) and the number of methyl groups added (mono-, di-, or trimethylation). For example, trimethylation of H3K4 (H3K4me3) is typically found at active gene promoters, whereas trimethylation of H3K27 (H3K27me3) is a hallmark of facultative heterochromatin and gene silencing. These methylation marks are dynamically written by histone methyltransferases (HMTs) and erased by histone demethylases (KDMs) (Barski et al., 2007; Cloos et al., 2008; Black et al., 2012; Hyun et al., 2017). Among the histone methyltransferase complexes, the COMPASS-like complexes containing members of the KMT2/MLL family, particularly *KMT2D* and *KMT2C*, catalyze the monomethylation of histone H3 at lysine 4 (H3K4me1), an activating mark at enhancers and promoters. Demethylases such as *KDM6A* (UTX) counterbalance this activity by removing the repressive H3K27me3 mark, thereby facilitating enhancer activation. Dysregulation of these COMPASS-related enzymes disrupts enhancer landscapes and transcriptional programs in multiple cancers, including PDAC (Jamali et al., 2024).

### 3.3 Chromatin remodeling

Chromatin remodeling includes dynamic exchanges that directly alter the structure and location of nucleosomes on DNA and thereby control the accessibility of genomic sites to transcription, replication, and DNA repair regulatory proteins. These exchanges are primarily carried out by large, multi-protein complexes known as ATP-dependent chromatin remodelers. These molecular devices drive nucleosome movement along DNA by the energy of ATP hydrolysis, evict them entirely, or swap canonical histones for histone variant ones and thereby open or close specific domains of chromatin (Eustermann et al., 2024). Chromatin remodelers are typically brought to a specific genomic location by interacting with sequence-specific DNA-binding proteins or by binding to specific histone modifications, all underscoring the intricate interplay between different levels of epigenetic regulation (Moshkin et al., 2012).

Among the major families of ATP-dependent remodelers, SWI/SNF, ISWI, CHD, and INO80, the SWI/SNF family, also known as the BRG1/BRM-associated factor (BAF) complex, is the most extensively characterized. This multi-subunit complex contains 12–15 components organized into canonical BAF, polybromo-BAF (PBAF), and non-canonical BAF (ncBAF) assemblies. The catalytic ATPases SMARCA4 (BRG1) or SMARCA2 (BRM) hydrolyze ATP to reposition nucleosomes, while structural and regulatory subunits such as ARID1A, ARID2, and SMARCB1 provide specificity and stability. By altering nucleosome occupancy at promoters and enhancers and cooperating with histone-modifying enzymes, SWI/SNF complexes establish chromatin states that facilitate or repress gene transcription, thereby playing critical roles in lineage specification, differentiation, and maintenance of cellular identity (Tsuda et al., 2021).

Higher-order chromatin topology, shaped by CTCF- and cohesin-mediated loop domains, maintains enhancer–promoter insulation and lineage-specific transcriptional programs. Disruption of such architectural features or TAD boundaries contributes to oncogenic enhancer hijacking and aberrant gene expression (Flavahan et al., 2016; Hnisz et al., 2016; Rowley and Corces, 2018; Sultana et al., 2025).

### 3.4 Non-coding RNAs

For many years, the central dogma of molecular biology focused on DNA encoding RNA, which in turn encodes protein. However, it is now clear that a vast portion of the human genome is transcribed into RNA molecules that do not code for proteins. These are known as non-coding RNAs (ncRNAs), and they constitute a diverse and functionally critical class of regulatory molecules. Among the most studied ncRNAs in the context of epigenetics and cancer are microRNAs (miRNAs) and long non-coding RNAs (lncRNAs) (Loganathan and Doss C, 2023).

miRNAs are small, evolutionarily conserved RNA molecules, typically around 17-25 nucleotides in length. They primarily function in post-transcriptional gene regulation. miRNAs usually bind to complementary sequences in the 3′ untranslated region (3′ UTR) of target messenger RNAs (mRNAs), leading to either the degradation of the mRNA or the repression of its translation into protein. A single miRNA can target hundreds of different mRNAs, and a single mRNA can be targeted by multiple miRNAs, creating complex regulatory networks that fine-tune gene expression (Bartel, 2009; Fabian et al., 2010; Rani and Sengar, 2022).

lncRNAs are a much more heterogeneous class of ncRNAs, operationally defined as being longer than 200 nucleotides (Mattick et al., 2023). They exhibit a wide array of molecular mechanisms and can regulate gene expression at multiple levels, chromatin organization, transcription, and post-transcriptional processing. lncRNAs can act as scaffolds by bringing together multiple proteins (e.g., chromatin-modifying enzymes) to form functional complexes (Mattick et al., 2023). They can act as guides by directing these complexes to specific genomic locations by base-pairing with DNA or RNA. For example, some lncRNAs interact with DNMTs or histone-modifying complexes like Polycomb Repressive Complex 2 (PRC2), guiding them to target gene promoters to mediate epigenetic silencing (Peschansky and Wahlestedt, 2014). Their expression can reflect specific cellular states or developmental processes (Fatica and Bozzoni, 2014). Dysregulation of both miRNA and lncRNA expression is a common feature of cancer, including pancreatic cancer, where they can function as oncogenes (promoting cancer) or tumor suppressors (inhibiting cancer) by influencing a wide range of cellular processes.

The various epigenetic mechanisms; DNA methylation, histone modifications, chromatin remodeling, and ncRNA-mediated regulation, do not function as isolated entities. Instead, they form a highly sophisticated and interconnected regulatory network. There is extensive crosstalk between these pathways. For instance, specific histone modifications can serve as docking sites for DNA methyltransferases, thereby directing DNA methylation to particular genomic regions. Conversely, DNA methylation patterns can influence histone modifications. lncRNAs, as mentioned, play a crucial role in guiding epigenetic modifying complexes (both DNMTs and histone modifiers) to their targets. Chromatin remodeling complexes are often recruited by, and work in concert with, histone-modifying enzymes. This intricate web of interactions allows for precise and robust control of gene expression. However, it also means that a disruption in one component of this network can have far-reaching and cascading effects on the entire epigenome, potentially leading to the widespread gene dysregulation characteristic of cancer. Understanding these interconnections is vital not only for deciphering the basic biology of gene regulation but also for predicting the outcomes of therapeutic interventions that target specific epigenetic pathways and for designing more effective, rational combination strategies.

A summary of key epigenetic mechanisms and their general effects on gene expression is presented in Table 1.

**TABLE 1:** Overview of key epigenetic mechanisms.

Mechanism	Key enzymes/Players	General impact on gene expression
DNA Methylation	DNMTs (DNMT1, DNMT3A, DNMT3B), TETs	Typically silencing (if at promoter)
Histone Acetylation	Histone Acetyltransferases (HATs), Histone Deacetylases (HDACs)	Typically activation (HATs)/repression (HDACs)
Histone Methylation	Histone Methyltransferases (HMTs), Histone Demethylases (KDMs)	Context-dependent activation or repression
Histone Ubiquitination	E3 Ubiquitin Ligases, Deubiquitinating Enzymes (DUBs)	Context-dependent effects on transcription/chromatin
miRNA	Dicer, Argonaute (AGO) proteins/RISC complex	Post-transcriptional gene silencing (mRNA degradation or translational repression)
lncRNA	RNA Polymerase II, various protein complexes (e.g., PRC2, DNMTs)	Transcriptional/post-transcriptional regulation via diverse mechanisms (scaffold, guide, decoy, signal)

## 4 Epigenetic dysregulation in pancreatic cancer

In pancreatic cancer, the finely tuned epigenetic regulation loses its harmony, leading to a discordant symphony of gene expression that fuels the initiation, progression, and resilience of the disease. This dysregulation affects all major epigenetic mechanisms as shown in Figure 1.

**FIGURE 1:**
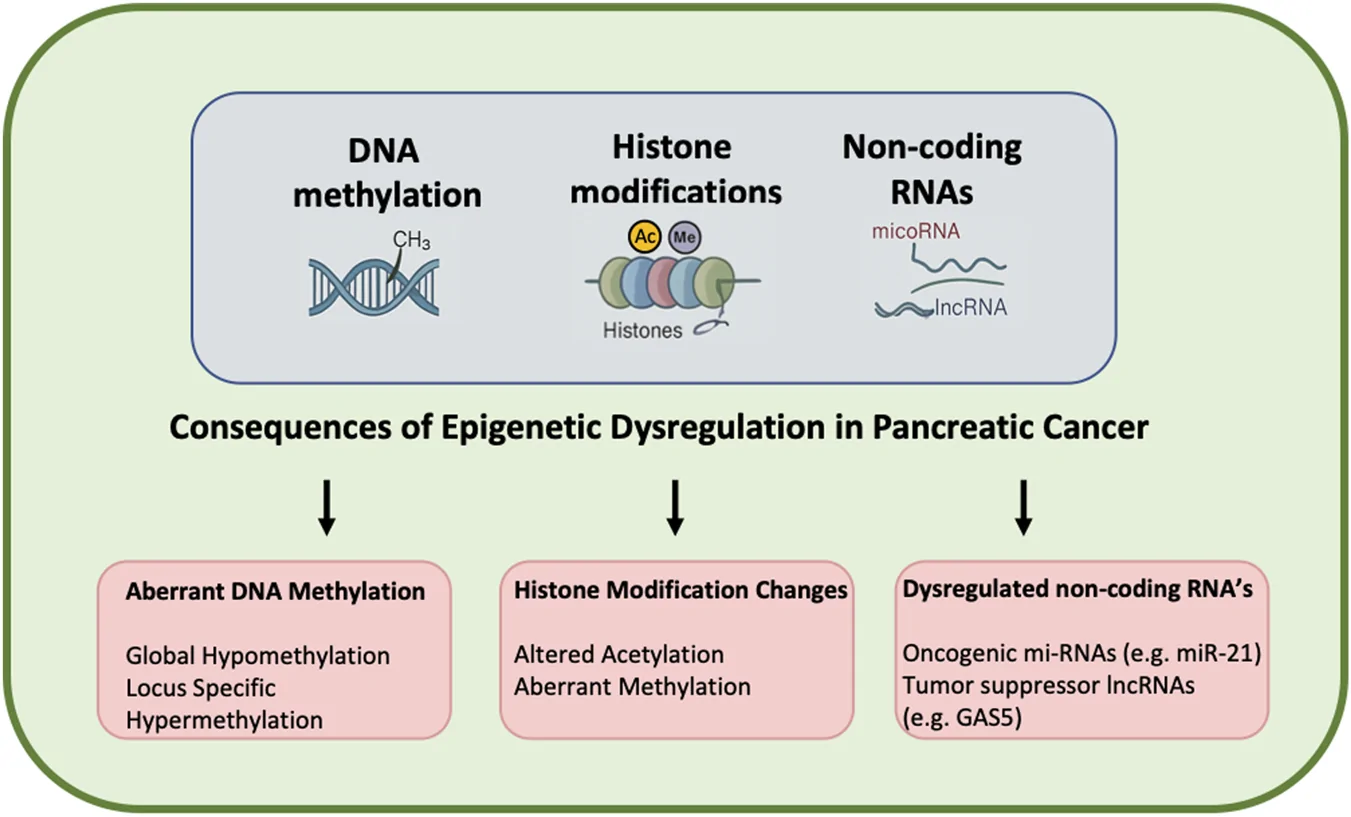
Epigenetic dysregulation in pancreatic cancer. This schematic illustrates the role of epigenetic dysregulation in the initiation and progression of pancreatic cancer. Epigenetic changes comprising DNA methylation, histone modification, and non-coding RNA function influence gene expression independently of the underlying DNA sequence. In pancreatic cancer, tumor suppressor gene (TSG) promoter hypermethylation leads to silencing of tumor suppressor genes, while global hypomethylation can lead to oncogene activation. Histone modifications, including abnormal methylation and acetylation patterns, also disrupt chromatin structure and regulation of gene expression, resulting in tumorigenesis. Non-coding RNAs (e.g., microRNAs and long non-coding RNAs) also modulate oncogenic and tumor suppressor pathways.

### 4.1 Aberrant DNA methylation in PDAC

The DNA methylation landscape in pancreatic cancer cells is profoundly distorted when compared to that of normal pancreatic tissue. Two major types of aberrations are typically observed. First is global hypomethylation; a widespread decrease in overall DNA methylation levels across the genome. This can lead to chromosomal instability, activation of transposable elements, and inappropriate expression of oncogenes that are normally kept silent. Second is locus-specific hypermethylation; an increase in DNA methylation at specific genomic regions, most notably at CpG islands located in the promoter regions of tumor suppressor genes (TSGs). This targeted hypermethylation results in the transcriptional silencing of these critical genes, effectively removing cellular brakes on proliferation and survival.

The silencing of TSGs through promoter hypermethylation is a frequent and crucial event in pancreatic cancer development. A classic example is the *CDKN2A* gene (also known as *p16INK4a*), a key cell cycle regulator. Hypermethylation of the *CDKN2A* promoter leads to its silencing, contributing to uncontrolled cell division and is commonly observed in PDAC (Tang et al., 2015). Numerous other TSGs are also targets of epigenetic silencing in pancreatic cancer. For instance, hypermethylation of *SFRP1* (Secreted Frizzled Related Protein 1), and *NPTX2* (Neuronal Pentraxin 2) has been reported and is associated with a poorer prognosis for patients (Bu et al., 2008; Zhang et al., 2012).

Conversely, hypomethylation can also contribute to oncogenesis by leading to the aberrant activation of proto-oncogenes or genes involved in invasion and metastasis. These changes in DNA methylation are not random occurrences but are integral to the neoplastic process, driving tumor initiation, facilitating progression through various stages, and enabling the acquisition of malignant characteristics.

### 4.2 Dysregulated histone modifications in PDAC

The “histone code,” which dictates chromatin structure and gene accessibility, is frequently misinterpreted or rewritten in pancreatic cancer cells, leading to widespread dysregulation of gene expression programs that favor malignant growth, invasion, and survival.

#### 4.2.1 Altered acetylation

The balance between histone acetylation and deacetylation is often skewed. Increased activity or overexpression of histone deacetylases (HDACs), such as HDAC1 and HDAC2, is a common finding in pancreatic cancer (Giaginis et al., 2015; Xiang et al., 2022). This leads to reduced levels of histone acetylation, resulting in a more condensed chromatin structure and the repression of critical genes, including tumor suppressors like p27 and p53, and cell adhesion molecules like E-cadherin. The downregulation of E-cadherin, for example, is a hallmark of Epithelial-to-Mesenchymal Transition (EMT), a process crucial for cancer cell invasion and metastasis (Nowak and Bednarek, 2021). Histone acetyltransferases (HATs), such as p300/CBP-associated factor (PCAF), also exhibit altered activity or expression and play complex roles. For instance, p300 helps maintain the expression of GATA6, a transcription factor that inhibits dedifferentiation and EMT in PDAC (Zhong et al., 2022). PCAF, while promoting transcription, can also be involved in pathways that enhance pancreatic cancer cell motility (Li et al., 2020).

#### 4.2.2 Aberrant methylation

Specific histone methylation marks, which can be either activating or repressive, are also profoundly dysregulated. A notable example is the loss of trimethylation on histone H3 lysine 9 (H3K9me3), a mark generally associated with heterochromatin and gene silencing. Loss of H3K9me3 has been linked to increased metastatic progression in pancreatic cancer (McDonald et al., 2017).

In addition to global and site-specific alterations in histone methylation, recurrent mutations or loss of *KMT2D*, *KMT2C*, and *KDM6A* represent some of the most frequent chromatin-modifying events in PDAC. *KDM6A* loss impairs enhancer function and cooperates with oncogenic *KRAS* to promote squamous-like differentiation and tumor progression (Andricovich et al., 2018), whereas *KMT2D* deficiency perturbs enhancer integrity, compromises immune-signaling pathways, and sensitizes tumors to immune-checkpoint blockade (Wang et al., 2020). Collectively, these findings underscore that aberrations in COMPASS-like complexes fundamentally reshape the enhancer landscape of PDAC (Jamali et al., 2024).

Enhancer of Zeste Homolog 2 (EZH2) is the catalytic subunit of the Polycomb Repressive Complex 2 (PRC2), responsible for depositing the repressive H3K27me3 mark. EZH2 is frequently overexpressed in PDAC and its high activity is associated with poorer patient outcomes, increased cancer cell stemness, and the promotion of EMT (Chang and Hung, 2012). Other histone methyltransferases (e.g., PRMT1, PRMT5, SETD2, KMT5A) and histone demethylases (e.g., KDM2B, KDM3A, KDM4B, KDM5A, KDM6A) are also found to be dysregulated in pancreatic cancer, impacting processes like EMT, cell proliferation, and survival by altering the methylation status of specific histone residues and thereby modulating target gene expression (Tzatsos et al., 2013; Andricovich et al., 2018; Niu et al., 2020; Hou et al., 2021; Li H. et al., 2021; Ku et al., 2024; Schneider et al., 2025).

### 4.3 Dysregulated chromatin remodeling in PDAC

The SWI/SNF (BAF) chromatin-remodeling complex is recurrently altered in approximately 14% of pancreatic ductal adenocarcinomas (PDACs), most frequently involving ARID1A, SMARCA4 (BRG1), and SMARCB1 (Tsuda et al., 2021). These alterations disrupt nucleosome positioning and transcriptional control, driving dedifferentiation, epithelial-mesenchymal transition (EMT), and undifferentiated tumor morphology. Functional studies demonstrate that ARID1A and SMARCB1 act as tumor suppressors whose loss accelerates pancreatic tumorigenesis, whereas SMARCA4/BRG1 exhibits context-dependent functions, suppressing initiation of ductal lesions but promoting acinar-derived neoplasia at later stages. Together, these findings implicate defective SWI/SNF-mediated chromatin remodeling as a key epigenetic driver of PDAC progression and therapeutic resistance (Tsuda et al., 2021).

### 4.4 Dysregulated non-coding RNAs in PDAC

The expression levels of a multitude of non-coding RNAs, particularly miRNAs and lncRNAs, are significantly altered in pancreatic cancer. These dysregulated ncRNAs can function as oncogenes (onco-miRs or onco-lncRNAs) if they promote cancer, or as tumor suppressors (ts-miRs or ts-lncRNAs) if their loss contributes to cancer development. They play pivotal roles in virtually all aspects of pancreatic cancer biology, including initiation, proliferation, invasion, metastasis, EMT, the maintenance of cancer stem cell (CSC) properties, and the development of chemoresistance.

#### 4.4.1 Oncogenic ncRNAs

Several ncRNAs are upregulated in pancreatic cancer and contribute to its aggressive phenotype. miR-21 is one of the most consistently overexpressed miRNAs in PDAC. miR-21 promotes tumor growth and resistance to chemotherapy by repressing the expression of multiple tumor suppressor genes, including *PTEN*, *PDCD4*, and *TPM1* (Stefanoudakis et al., 2025). miR-155 is also frequently upregulated, it enhances proliferation and invasion, in part by targeting *SOCS1* (Stefanoudakis et al., 2025).

HOTAIR is highly expressed in pancreatic cancer and promotes cell proliferation, invasion, and metastasis. It functions by interacting with epigenetic complexes like PRC2 (to mediate H3K27me3) and LSD1/CoREST/REST (to mediate H3K4 demethylation), thereby altering gene transcription programs (Yang et al., 2017). Other oncogenic lncRNAs include HOTTIP, H19, PVT1, MALAT-1, and HULC, each contributing to malignancy through various mechanisms such as activating Wnt/β-catenin or PI3K/AKT signaling pathways, or promoting EMT (De Martino et al., 2021).

#### 4.4.2 Tumor suppressive ncRNAs

Conversely, many ncRNAs that normally function to restrain cell growth or promote differentiation are downregulated or lost in pancreatic cancer. Members of the let-7 miRNA family are often reduced in pancreatic cancer. Their reinstatement can inhibit cell proliferation, partly by targeting the *KRAS* oncogene (Karmakar et al., 2019). Downregulation of miR-34a, a tumor-suppressive miRNA that targets Notch signaling and restrains cancer stem cell self-renewal, is common. Restoring its expression can impede cancer progression by inhibiting Notch signaling, which is important for CSC self-renewal (Li W. J. et al., 2021). lncRNA GAS5 (Growth Arrest-Specific 5) is often downregulated and acts as a tumor suppressor by influencing cell proliferation and cell cycle regulation (Zhou and Chen, 2020). Numerous other miRNAs (e.g., miR-494, miR-216a, miR-148a, miR-192, miR-203, miR-96, and miR-217) and lncRNAs have been identified as tumor suppressors whose diminished expression contributes to pancreatic cancer (Sharma et al., 2021).

Key dysregulated epigenetic factors and their downstream consequences in pancreatic cancer are summarized in Table 2.

**TABLE 2:** Dysregulated epigenetic factors in pancreatic cancer.

Epigenetic factor	Mechanism	Dysregulation in PDAC	Functional consequence	References
DNMT1	DNA Methylation (writer)	Overexpression	Silencing of TSGs, promoting proliferation, invasion, stemness	Tang et al. (2015)
*p16INK4a* (*CDKN2A*) promoter	DNA methylation	Hypermethylation	Silencing of *p16INK4a* TSG, leading to uncontrolled cell proliferation	Tang et al. (2015)
p300/CBP	Histone acetyltransferase (writer)	Aberrant expression/activity	p300 maintains GATA6 (inhibits EMT)	Zhong et al. (2022)
HDAC1/HDAC2	Histone Deacetylase (eraser)	Increased activity/Overexpression	Repression of TSGs (e.g., p27, p53), E-cadherin; promoting proliferation, invasion, EMT	Giaginis et al. (2015)
H3K9me3	Histone methylation	Global loss/Alterations	Associated with metastatic progression	McDonald et al. (2017)
EZH2	Histone methylransferase (writer)	Overexpression/Increased H3K27me3	Repression of TSGs (e.g., E-cadherin), promoting stemness, EMT, poor prognosis	Chang and Hung (2012)
PRMT1/PRMT5	Histone methyltransferase (writer)	Overexpression	Promote proliferation, EMT (PRMT5 via EGFR/AKT/β-catenin)	Lee et al. (2021), Ku et al. (2024)
miR-21	miRNA	Overexpression	Represses TSGs (PTEN, PDCD4), enhances growth, chemoresistance	Stefanoudakis et al. (2025)
miR-34a	miRNA	Downregulation	Tumor suppressive; its loss contributes to progression (e.g., via Notch signaling)	Li et al. (2021b)
lncRNA HOTAIR	Chromatin scaffold	Overexpression	Promotes proliferation, invasion, EMT, chemoresistance; interacts with PRC2, LSD1	Yang et al. (2017)
lncRNA GAS5	Tumor suppressor lncRNA	Downregulation	Tumor suppressive; its loss linked to increased proliferation	Zhou and Chen (2020)

### 4.5 Epigenetic drivers of heterogeneity, metastasis, and immune evasion

Epigenetic alterations are inherent drivers of intra-tumoral heterogeneity in PDAC. Such heterogeneity, i.e., having heterogeneous cancer cell populations within a tumor, allows the entire tumor to adapt to shifting environments, become more invasive, gain metastatic capability, and develop drug resistance. Epigenetic plasticity supports the capacity of cancer cells to switch between different phenotypic states, resulting in such heterogeneity (Flavahan et al., 2017).

Epithelial-to-Mesenchymal Transition (EMT) is a critical cell program that allows epithelial cancer cells to lose cell-cell adhesion, acquire migratory and invasive potential, and disseminate to remote sites, forming metastases. Epigenetic reprogramming causes induction and maintenance of the EMT phenotype. Epigenetic silencing of epithelial markers (e.g., E-cadherin) and activation of mesenchymal markers are part of this process (Kalluri and Weinberg, 2009). Furthermore, epigenetic mechanisms are important in facilitating PDAC cells to escape immune detection and killing by the host immunity. Cancer cells can epigenetically repress the expression of genes that code for Major Histocompatibility Complex (MHC) molecules that are necessary for presenting tumor antigens to T cells. They can epigenetically enhance the expression of immune checkpoint molecules such as PD-L1 (Programmed Death-Ligand 1) (Chen et al., 2020).

### 4.6 Cross-talk between driver mutations and epigenetic programs

Pancreatic cancer is characterized by a constellation of recurrent genetic changes in a subset of crucial driver genes. Activating mutations within the *KRAS* oncogene are found in over 90% of PDACs, an initiating and obligatory event in its development (Buscail et al., 2020). Inactivating mutations or loss-of-function of tumor suppressor genes such as *TP53* (*p53*), *CDKN2A* (coding for p16INK4a and p14ARF), and *SMAD4* are also highly frequent and typically occur at later stages of tumor evolution (Sun et al., 2020).

These specifying genetic events, while not operating *in vacuo*, are in a running and bidirectional conversation with the epigenetic machinery. Oncogenic KRAS, for example, is not just a signaling molecule but can be a master regulator that induces global epigenetic reprogramming. Activated KRAS signaling can remodel DNA methylation patterns and histone modification landscapes, thereby remodeling the expression of numerous downstream genes to promote cell growth, survival, and metabolic adaptation (Mathison et al., 2021). *TP53* mutations also influence epigenetic regulation. Wild-type p53 plays a role in epigenetic maintenance; p53 loss or mutation leads to epigenetic dysregulation, such as abnormal DNA methylation and changed chromatin accessibility at target gene loci. Mutant p53 proteins even gain new oncogenic functions that involve association with epigenetic regulators to promote tumor growth (Freed-Pastor and Prives, 2012).

Conversely, epigenetic mechanisms could influence the function or expression of such key cancer genes more directly. For instance, the tumor suppressor *CDKN2A* can be silenced by genetic loss or mutation as well as by epigenetic silencing as a result of promoter hypermethylation, a common feature of pancreatic cancer (Winter et al., 2006). Loss of *SMAD4*, a key component of the TGF-β pathway, is also a common event, and its functional impacts are influenced by the epigenetic status of the cell (Wang Q. et al., 2023).

This complex interplay means that genetic mutations can trigger cascades of epigenetic alterations and that these epigenetic alterations have the ability to regulate the functional impact of the genetic mutations or even to propel oncogenic programs on their own by repressing unmutated genes. The numerous molecular subtypes of PDAC identified are often characterized by distinct patterns of such genetic and epigenetic alterations, reflective of the composite nature of these alterations in determining tumor biology.

The presence of specific driver mutations, such as in *KRAS* or *TP53*, may possibly alter the cellular state in a way that cancer cells become uniquely dependent on certain epigenetic pathways for their continued survival or proliferation. This concept suggests that such mutations would have the capacity to create “epigenetic vulnerabilities”. That is, a cell carrying an oncogenic *KRAS* mutation could reprogram its epigenetic landscape to sustain the rates of proliferation and metabolic activity induced by KRAS signaling. Such reprogramming could involve overreliance on a particular histone methyltransferase or chromatin reader protein. If such a dependency is established, then inhibiting that specific epigenetic enzyme or reader protein could be selectively lethal to the *KRAS*-mutant cancer cells and would not harm normal cells (cells that lack the *KRAS* mutation and thus the induced dependency) comparatively. This principle, that has been referred to as synthetic lethality, offers a very appealing means of developing targeted therapy to exploit the cancer cell’s genetic profile in order to guide the selection of epigenetic drugs (Yang et al., 2019). Such investigations into synthetic lethal interaction between genetic mutation and epigenetic mechanism are a new research area to identify new therapeutic targets tailored to the patient’s own tumor molecular signature.

## 5 Epigenetic biomarkers for pancreatic cancer

The extensive and often early epigenetic alterations in pancreatic cancer have led to significant research into their utility as clinical biomarkers. They can revolutionize the detection, diagnosis, and treatment of pancreatic cancer, addressing significant unmet needs in a late-diagnosed disease with limited prognostic aids.

### 5.1 Early detection and diagnostic epigenetic markers

One of the most compelling possible applications of epigenetic markers is early detection and diagnosis. Because epigenetic changes can occur before clinical malignancy or even during the earliest stages of cancer formation, they offer a window for detecting the disease when it is perhaps more curable. Figure 2 summarizes key epigenetic biomarkers and their clinical applications in pancreatic cancer.

**FIGURE 2:**
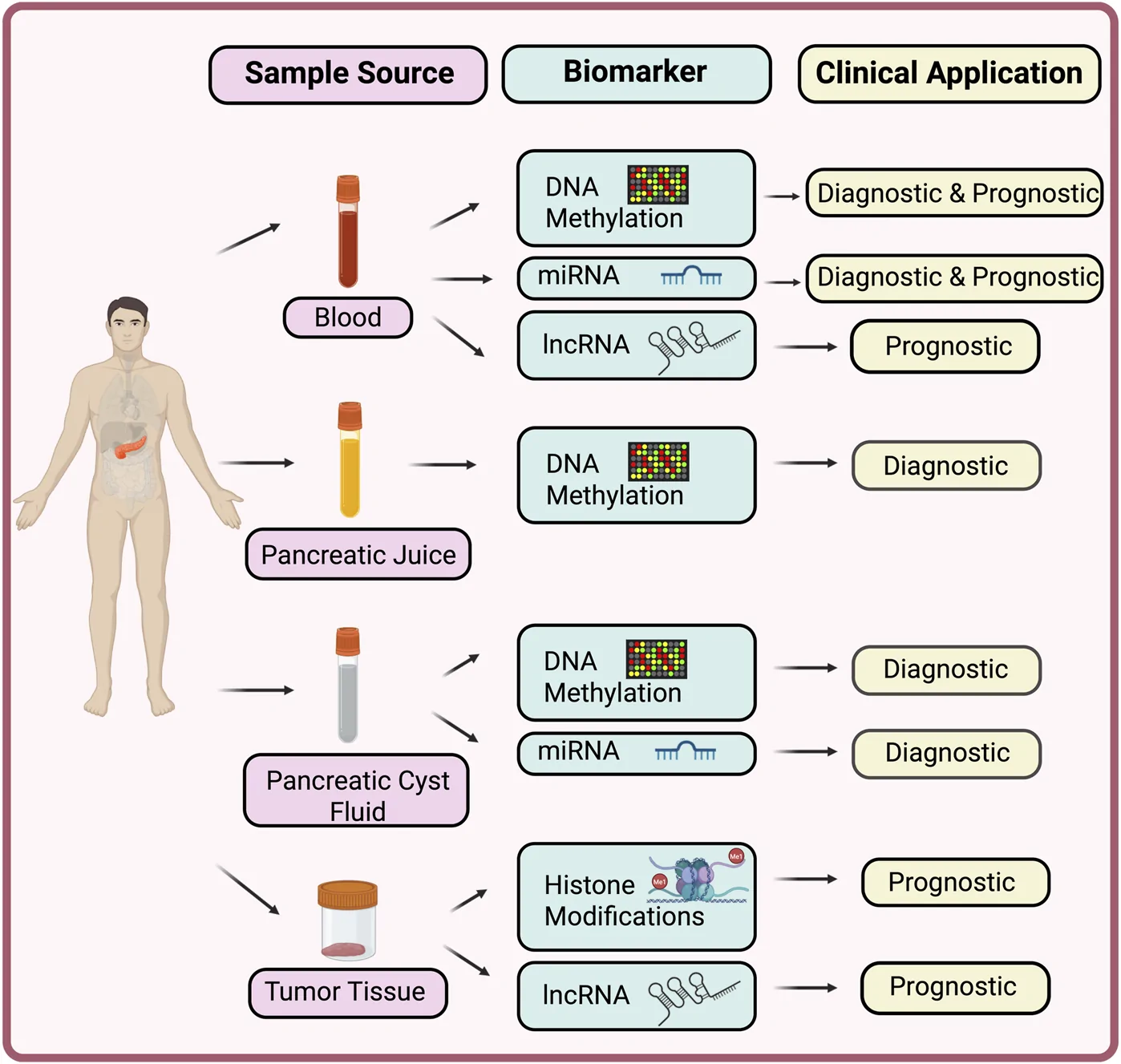
Epigenetic biomarkers in pancreatic cancer. This schematic illustrates the range of epigenetic biomarkers detectable from different sample sources in pancreatic cancer. Simple blood-based assays for DNA methylation markers and non-coding RNAs can provide both diagnostic and prognostic information, enabling minimally invasive detection of disease at an early stage. Pancreatic-juice analyses, focused on DNA methylation profiling, offer enhanced diagnostic accuracy. Pancreatic cyst fluid, also serves as a valuable source of both methylated DNA markers and miRNA signatures that can distinguish high-grade dysplasia or malignant cysts from low-risk lesions. Tissue-based assays, including histone-modification profiling and lncRNA characterization, yield deeper prognostic insights and aid in the molecular stratification of patient outcomes.

#### 5.1.1 DNA methylation markers

Aberrant DNA methylation patterns, and more particularly the hypermethylation of specific gene promoters, are stable and detectable alterations. They are detectable not merely in tumor tissue obtained by biopsy but also in less invasive media like pancreatic juice, pancreatic cyst fluid and, significantly, in circulating cell-free DNA (cfDNA) in plasma or serum. This has led to the promise of “liquid biopsy” approaches. Methylated DNA marker (MDM) panels are in development and testing. For example, a study identified a panel of methylated BNC1 (Basonuclin 1) and ADAMTS1 (ADAM Metallopeptidase With Thrombospondin Type 1 Motif 1) whose presence in the serum had high sensitivity (81%) and specificity (85%) for the identification of pancreatic cancer (Yi et al., 2013). Yet another highly promising DNA methylation signature includes six specific CpG sites of the *PRKCB* (*Protein Kinase C Beta Type*) gene. In one study, such a signature correctly segregated PDAC from chronic pancreatitis (a common diagnostic challenge) in tissue samples and plasma-derived cfDNA 100% of the time (Wu et al., 2023). Furthermore, the methylation status of mucin genes detected in pancreatic juice and methylated DNA marker panels identified in cyst fluid obtained via endoscopic ultrasound–guided fine-needle aspiration (EUS-FNA) have also shown diagnostic value, with studies demonstrating the ability to distinguish high-grade dysplasia and early malignancy from benign cysts (Yokoyama et al., 2014; Majumder et al., 2019; Engels et al., 2025).

#### 5.1.2 Histone modification markers

While histone modification detection in liquid biopsies is technically more challenging and not as developed as DNA methylation analysis, certain patterns of histone modifications in tumor tissue are able to differentiate tumor tissue from normal pancreatic tissue and could potentially be of interest in diagnosis.

#### 5.1.3 Non-coding RNA markers

Aberrant miRNAs and lncRNAs are also emerging as significant diagnostic biomarkers. Specific miRNAs (e.g., miR-21, miR-155, miR-196a, miR-210) and lncRNAs (e.g., HOTAIR) have altered levels in the blood (plasma, serum) or other body fluids (e.g., urine exosomes or extracellular vesicles) of pancreatic cancer patients compared to controls or those with benign pancreatic disease (Bravo-Vázquez et al., 2022). In addition, pancreatic cyst fluid has been recognized as a valuable source for miRNA-based biomarker detection, with studies showing elevated levels of miR-21, miR-221, miR-155, and miR-210 in cysts harboring high-grade dysplasia or invasive carcinoma (Farrell et al., 2013; Shirakami et al., 2021). Broader miRNA-profiling analyses of cyst fluid have identified diagnostic panels that effectively distinguish malignant or high-risk intraductal papillary mucinous neoplasms (IPMNs) from benign cysts (Matthaei et al., 2012; Wang et al., 2015; Utomo et al., 2016). Integrated molecular studies further support the clinical utility of miRNA panels derived from pancreatic cyst fluid (Maher et al., 2025). Multimarker panels of miRNAs are shown to be more diagnostic than single markers (Matthaei et al., 2012; Utomo et al., 2016). The establishment of liquid biopsy techniques for their identification is a particularly significant milestone. The ability to identify cancer-type epigenetic patterns in easily accessible body fluids like blood, pancreatic juice, pancreatic cyst fluid or urine potentially opens the way for non-invasive or low-invasive screening regimens for high-risk populations and more accurate early diagnosis.

### 5.2 Prognostic epigenetic markers

Beyond diagnosis, epigenetic markers can furnish valuable prognostic information, helping to forecast the likely course of the disease, its aggressiveness, and patient survival outcomes.

#### 5.2.1 DNA methylation

Specific DNA methylation patterns have been linked to prognosis. For instance, the hypermethylation of genes such as *SFRP1* and *NPTX2* in tumor tissue is associated with a poorer prognosis in PDAC patients (García-Ortiz et al., 2023). Comprehensive DNA methylome profiling can also stratify patients into distinct risk groups with differing survival expectancies (Yong et al., 2016).

#### 5.2.2 Histone modifications

The overall levels or specific patterns of histone modifications within tumor cells can also predict patient outcomes. Studies have demonstrated that low cellular levels of certain histone marks, such as H3K4 dimethylation (H3K4me2), H3K9 dimethylation (H3K9me2), or H3K18 acetylation (H3K18ac), in resected pancreatic adenocarcinoma tissues are independent predictors of poor survival (Manuyakorn et al., 2010; Watanabe et al., 2012).

#### 5.2.3 Non-coding RNAs

The expression levels of various miRNAs and lncRNAs in tumor tissue or circulation have been correlated with patient prognosis. For example, high circulating levels of miR-21, miR-155, or miR-196a, or lncRNAs like HOTAIR, are often associated with shorter overall survival and increased risk of disease recurrence. Conversely, higher expression of tumor-suppressive miRNAs like miR-192 or lower expression of lncRNAs like LINC01111 may indicate a better prognosis (Marin et al., 2023).

Table 3 summarizes key epigenetic biomarkers in pancreatic cancer and their diagnostic and prognostic relevance.

**TABLE 3:** Epigenetic biomarkers in pancreatic cancer.

Biomarker type	Biomarker/Gene	Sample source	Role	Clinical relevance	Key reference
DNA Methylation	*BNC1*, *ADAMTS1*	cfDNA (plasma/serum)	Diagnostic – distinguishes PDAC from controls	Combined methylation panel, 81% sensitivity, 85% specificity for PDAC	Yi et al. (2013)
DNA Methylation	*PRKCB* CpG signature	cfDNA and tissue	Diagnostic – separates PDAC from chronic pancreatitis	100% accuracy in study cohort	Wu et al. (2023)
DNA Methylation	*SFRP1*, *NPTX2* promoter hypermethylation	Tumor tissue	Prognostic – associated with poor survival	Independent predictors of outcome	García-Ortiz et al. (2023)
DNA Methylation	Mucin gene methylation	Pancreatic juice	Diagnostic marker for early PDAC	Distinguishes neoplasms via DNA methylation analysis	Yokoyama et al. (2014)
DNA Methylation	Methylated DNA Markers (MDM) panel	Pancreatic cyst fluid	Diagnostic	Discriminates high grade dysplasia from low grade dysplasia	Majumder et al. (2019)
Histone Modifications	Low H3K4me2, H3K9me2, H3K18ac levels	Tumor tissue	Prognostic – poor survival after resection	Independent predictor of poor survival	Manuyakorn et al., 2010; Watanabe et al., 2012
miRNA	miR-21, miR-155, miR-196a, miR-210	Plasma/serum/exosomes	Diagnostic and Prognostic	Elevated levels correlate with poor survival	Bravo-Vázquez et al., 2022; Stefanoudakis et al., 2025
miRNA	miR-711, miR-3679-5p, miR-6126, miR-6780b-5p, miR-6798-5p, miR-6879-5p	Pancreatic cyst fluid	Diagnostic – detects malignant transformation in IPMN	Comprehensive profiling identified six miRNAs significantly upregulated in cyst fluid from IPMC vs. IPMA	Shirakami et al. (2021)
miRNA	Nine-miRNA signature (miR-18a, miR-24, miR-30a-3p, miR-92a, miR-99b, miR-106b, miR-142-3p, miR-342-3p, miR-532-3p)	Pancreatic cyst fluid	Diagnostic – distinguishes high-grade IPMN and other high-risk cysts from low-risk lesions	Nine-miRNA model identified high-risk cysts with 89% sensitivity and 100% specificity	Matthaei et al. (2012)
lncRNA	HOTAIR	Tissue/plasma	Prognostic – high levels predict shorter survival	Indicator of EMT and chemoresistance	Bravo-Vázquez et al., 2022, Yang et al., 2017
lncRNA	LINC01111	Tumor tissue/plasma	Prognostic – loss linked to poor outcome	Tumor suppressive	Marin et al. (2023)

### 5.3 Predictive epigenetic markers for therapeutic response

One of the most significant objectives in oncology is to personalize treatment by selecting therapies with the greatest likelihood of benefiting an individual patient. Epigenetic markers represent attractive predictive biomarkers with the potential to foresee a patient’s response to a specific treatment, thereby guiding therapeutic decisions.

For instance, one study found that tumor tissue cellular levels of the histone modifications H3K4me2 and H3K9me2 were predictive of survival outcome in the particular situation of patients with resectable pancreatic adenocarcinoma treated with adjuvant chemotherapy with fluorouracil, but not in patients treated with gemcitabine (Manuyakorn et al., 2010). This suggests that such markers could be helpful to aid clinicians in choosing the most appropriate adjuvant chemotherapy regimen for specific groups of patients. Recent work also indicates that transcriptional subtypes defined by GATA6 expression may predict response to gemcitabine-based therapy, with GATA6-negative tumors deriving greater benefit (Guenther et al., 2025). Given that GATA6 functions with the histone acetyltransferases p300/CBP as transcriptional co-activators, alterations in p300/CBP activity could theoretically modulate chromatin states and influence chemotherapy sensitivity, although direct evidence in PDAC is still limited (Guenther et al., 2025).

As epigenetic therapies themselves become more commonly integrated into pancreatic cancer therapeutic regimens, the identification of robust epigenetic biomarkers of sensitivity or resistance to these novel agents will be crucial in order to optimize their usefulness.

Notably, loss of *KDM6A* has been reported to confer heightened sensitivity to BET inhibitors and to potentiate the cytotoxic effects of HDAC inhibitors, highlighting its potential as a predictive biomarker for responsiveness to epigenetic-drug therapy (Andricovich et al., 2018; Watanabe et al., 2019).

While the discipline is filled with promising epigenetic biomarker candidates, there exists a wide chasm between their discovery in the laboratory and their implementation in daily clinical practice.

The journey of a biomarker from discovery to widespread clinical application is long and treacherous. It needs to go through rigorous clinical validation in big, independent, and diverse patient groups to determine its sensitivity, specificity, and overall clinical utility. Furthermore, tests for the detection of these epigenetic markers must be standardized, reproducible, inexpensive, and suitable for integration into existing clinical laboratory practice. This encompasses normalizing sample procurement, processing, analytical methods, and data interpretation. It is the focus of ongoing research to surmount these translational hurdles with a view to bringing the promise of epigenetic biomarker discovery to the patient.

## 6 Pharmacological targeting of the epigenome

The intrinsic dynamism and reversibility of epigenetic marks make a compelling case for developing therapeutic strategies that seek to “rewrite” or “correct” the aberrant epigenetic landscape that characterizes cancer cells. Unlike genetic mutations, which are permanent alterations of the DNA sequence, epigenetic marks can, in principle, be erased or rewritten by drug-like molecules.

### 6.1 The rationale: why epigenetic therapies hold promise

The central objective of epigenetic therapy is to control gene expression programs in cancer cells by focusing on enzymes and proteins responsible for establishing, stabilizing, or reading epigenetic marks. It could involve the re-expression of epigenetically silenced tumor suppressor genes, inhibition of oncogenic epigenetic pathways driven or sustained by epigenetic changes, or global reprogramming of the epigenetic state of cancer cells to render them less aggressive or susceptible to other therapeutic agents such as chemotherapy or immunotherapy. Due to the pervasive nature of epigenetic deregulation in PDAC, these “epi-drugs” constitute a novel and potentially effective treatment modality in an aggressive cancer with limited treatment choices.

### 6.2 Classes of epigenetic drugs

Several classes of epigenetic drugs are in various phases of preclinical and clinical development, targeting different components of the epigenetic machinery.

#### 6.2.1 DNA methyltransferase inhibitors (DNMTis)

DNMTis are designed to counteract the effect of aberrant DNA hypermethylation. The best-known DNMTis, such as azacitidine (5-azacytidine) and decitabine (5-aza-2′-deoxycytidine), are nucleoside analogs. Upon application during DNA replication, these analogs covalently trap DNMT enzymes (primarily DNMT1) that attempt to methylate them. Such trapping leads to the degradation of the DNMT enzymes and a passive loss of methylation patterns on subsequent DNA replication cycles, ultimately leading to DNA hypomethylation and re-expression of once-repressed tumor suppressor genes. DNMTis are currently FDA-approved for the treatment of certain hematological malignancies, e.g., myelodysplastic syndromes. They are actively under investigation in solid tumors, including PDAC, often in combination regimens. Drawbacks of current DNMTis include potential toxicities and the development of therapeutic resistance (Manuyakorn et al., 2010; Suraweera et al., 2025).

#### 6.2.2 Histone deacetylase inhibitors (HDACi)

Histone deacetylase inhibitors (HDACis) target the enzymes that eliminate acetyl groups from histone proteins. By inhibiting HDAC activity, these drugs induce an accumulation of acetylated histones, which has the effect of promoting a more open, transcriptionally permissive chromatin state. This can result in the reactivation of silenced tumor suppressor genes and other genes whose expression is helpful for anti-cancer activity, such as those with action in cell cycle arrest, apoptosis, and differentiation. Some of the examples of HDACis are vorinostat, panobinostat, entinostat, mocetinostat, belinostat, and romidepsin. Some of the HDACis are FDA-approved for the treatment of some hematologic cancers, particularly cutaneous T-cell lymphoma and multiple myeloma. In pancreatic cancer, HDACis are under investigation in clinical trials, most often with chemotherapy or other targeted therapies, since monotherapy has shown modest activity. As for DNMTis, side effects and the development of resistance are significant concerns (Suraweera et al., 2025).

#### 6.2.3 Histone methyltransferase inhibitors (HMTis)

HMTis are designed to block the activity of specific HMTs that “write” particular histone methylation marks. A prominent class of HMTis targets EZH2 (Enhancer of Zeste Homolog 2), the catalytic subunit of the PRC2 complex, which is responsible for trimethylating histone H3 at lysine 27 (H3K27me3), a mark associated with gene repression. EZH2 is frequently overexpressed in PDAC and its elevated activity is linked to poor prognosis, enhanced cancer stem cell properties, and the promotion of EMT by silencing key regulatory genes (Xu and Zhu, 2023). EZH2 inhibitors (EZH2i), such as tazemetostat (FDA-approved for certain lymphomas and sarcomas), aim to block EZH2’s methyltransferase activity, thereby reducing global H3K27me3 levels and leading to the derepression of PRC2 target genes (Julia and Salles, 2021). These agents have shown anti-tumor effects in preclinical PDAC models and are under clinical investigation. Inhibitors targeting other HMTs, such as PRMT5 (Protein Arginine Methyltransferase 5), like EZP015556, are also in clinical trials for various cancers, including PDAC (Lee et al., 2021).

#### 6.2.4 Bromodomain and extra-terminal Motif (BET) inhibitors

BET proteins, including BRD2, BRD3, and BRD4, are epigenetic “readers.” They recognize and bind to acetylated lysine residues on histone tails through their bromodomains. This binding often serves to recruit transcriptional machinery to gene promoters and enhancers, thereby activating the expression of target genes, including critical oncogenes like MYC and genes involved in inflammatory responses (Wang Z.-Q. et al., 2023). BET inhibitors (e.g., OTX015/birabresib, CPI-0610, JQ1, INCB057643) are small molecules that competitively bind to the bromodomains of BET proteins, displacing them from chromatin and thereby preventing the transcriptional activation of their target genes (Sarnik et al., 2021). By downregulating key oncogenic gene expression programs, BET inhibitors have demonstrated significant anti-tumor activity in preclinical models of PDAC, often by suppressing oncogenic signaling pathways and enhancing the efficacy of chemotherapy. Several BET inhibitors are currently being evaluated in clinical trials for various cancers, including PDAC.

### 6.3 Clinical trials in pancreatic cancer

A growing number of clinical trials are underway to evaluate the safety and efficacy of various epigenetic drugs in patients with pancreatic cancer, both as single agents and, more commonly, as part of combination regimens. While early enthusiasm was high, the results of monotherapy with epigenetic drugs in unselected PDAC patient populations have generally been modest. Contributing factors include: (i) pronounced intra-tumoral and stromal heterogeneity, which reduces uniform dependency on individual chromatin regulators; (ii) insufficient and/or transient epigenome reprogramming *in vivo* due to dose-limiting toxicities and challenges in achieving sustained pharmacodynamic target engagement; (iii) adaptive resistance through compensatory oncogenic and inflammatory circuits; and (iv) a historical lack of predictive biomarkers and on-treatment pharmacodynamic (PD) readouts to guide enrollment and dosing (Shorstova et al., 2021; Elrakaybi et al., 2022; Orlacchio et al., 2024). These issues are exemplified by the randomized Phase II adjuvant trial of oral azacitidine (CC-486) in resected high-risk PDAC, which did not prolong time-to-relapse (Heumann et al., 2022). Early clinical experience with HDAC inhibitors in PDAC has been primarily in combination, for example, vorinostat with capecitabine and radiation established feasibility/MTD rather than single-agent benefit, underscoring the limits of monotherapy (Chan et al., 2016).

Significant hurdles remain in the clinical development of epigenetic therapies for PDAC. These include the challenge of identifying patient subgroups most likely to respond to a particular epi-drug (biomarker discovery), managing potential on-target and off-target toxicities, understanding and overcoming mechanisms of intrinsic and acquired resistance, and navigating the complex and hostile tumor microenvironment of PDAC, which can limit drug delivery and efficacy. Nevertheless, each trial provides valuable learnings that inform the design of future studies, helping to optimize drug selection, dosing regimens, treatment schedules, and the choice of combination partners. Building on these insights, emerging BET- and EZH2-directed strategies, including dual BET/EP300 inhibition have been shown to suppress KRAS-driven transcriptional programs in PDAC models. They can also enhance antitumor immune responses, providing a strong rationale for combining these agents with chemotherapy or immune checkpoint blockade. In parallel, biomarker-guided and adaptive trial designs that incorporate tumor-tissue or cfDNA-based pharmacodynamic readouts are being developed to optimize treatment selection and dosing (Shorstova et al., 2021; Elrakaybi et al., 2022; Principe et al., 2022; Orlacchio et al., 2024). Collectively, these data support the use of combination regimens, patient stratification based on epigenetic dependencies, schedule optimization (“epigenetic priming”), and integrated PD monitoring as key steps toward translating the mechanistic promise of epigenetic therapy into clinically meaningful benefit in PDAC. Specific trials include those for DNMT inhibitors (azacytidine CC-486 in resected PDAC and guadecitabine with durvalumab), PRMT5 inhibitors (EZP015556), and HDAC inhibitors (ivaltinostat with gemcitabine and erlotinib) (Heumann et al., 2022; Jo et al., 2022; Orben et al., 2022).

### 6.4 Synergistic approaches: combining epigenetic drugs with chemotherapy and immunotherapy

The most promising path forward for epigenetic therapies in PDAC appears to be their use in combination with other established or emerging anti-cancer treatments.

#### 6.4.1 With chemotherapy

Epigenetic drugs have the potential to sensitize pancreatic cancer cells to conventional cytotoxic agents like gemcitabine, a cornerstone of PDAC chemotherapy (Baretti et al., 2019). They can achieve this by several mechanisms: altering chromatin structure to improve drug access to DNA targets, reactivating apoptotic pathways that were epigenetically silenced, reversing epigenetic changes that confer chemoresistance, or downregulating DNA repair mechanisms (Baretti et al., 2019). For example, preclinical studies and some early clinical trials have shown that combining HDAC inhibitors with gemcitabine can lead to enhanced anti-tumor effects.

#### 6.4.2 With immunotherapy

Pancreatic cancer is generally considered “cold” or non-immunogenic, exhibiting a poor response to immune checkpoint inhibitors (ICIs) like anti-PD-1/PD-L1 antibodies when used as monotherapy (Farhangnia et al., 2024). Epigenetic drugs are being actively explored as agents to “reprogram” or “remodel” the immunosuppressive TME of PDAC, thereby potentially “priming” the tumor for a more effective response to ICIs. Proposed mechanisms include enhancing the expression of tumor-associated antigens, increasing the infiltration of cytotoxic T cells and other beneficial immune cells into the tumor, reducing the populations or activity of immunosuppressive cells (like regulatory T cells or myeloid-derived suppressor cells), and reprogramming exhausted T cells. Both DNMT inhibitors and HDAC inhibitors have shown promise in preclinical models and early-phase clinical trials in their ability to modulate the immune landscape and synergize with ICIs (Ganji and Farran, 2022). Moreover, *KMT2D*-deficient tumors exhibit enhanced immunogenicity and an improved response to immune-checkpoint blockade, suggesting that mutational status of COMPASS-related genes may serve as a determinant of immunotherapy outcome (Wang et al., 2020; Jamali et al., 2024).

### 6.5 Epigenetics in overcoming therapeutic challenges

Drug resistance acquisition is one of the main causes of treatment failure in pancreatic cancer. The epigenetic plasticity inherent to cancer cells can be a powerful force for the development of resistance, not only to conventional chemotherapy and targeted treatments but also to epigenetic drugs themselves. Cancer cells can dynamically alter their epigenetic landscape to adapt to therapeutic stress, for example, by silencing genes associated with drug uptake or apoptosis, or by activating survival alternative pathways. Epigenetic treatments offer a potential means to counteract such resistance mechanisms by reversing the specific epigenetic marks that accompany resistance or by inducing synthetic lethality in resistant cell populations (Lazo, 2022; Liu et al., 2025). Still, epigenetic drug resistance itself can occur, for instance, because of mutations in the targeted epigenetic enzymes or because of compensatory alterations in other epigenetic pathways. Clarification of these resistance mechanisms is critical to the development of strategies to prevent, delay, or circumvent resistance to epigenetic and conventional therapies.

The effectiveness of epigenetic medications in PDAC is now known to depend not only on their short-term impact on cancer cells but significantly also on how they regulate the intricate TME. Such a broader effect would extend to activity on numerous stromal cell populations, such as pancreatic stellate cells and cancer-associated fibroblasts, and reduce possibly the compact desmoplastic response or reprogram them to assume a less tumor-promoting, if not tumor-inhibiting, phenotype. Besides, as described, these agents can significantly influence the immune cell infiltration into the tumor with the aim to re-balance the environment from an immunosuppressive to one that supports an effective anti-tumor immune response. This would involve a paradigm where epigenetic drugs are not merely viewed as short-term anti-cancer medicines but as “TME modulators.” Consequently, their effectiveness may rely on their ability to favorably reshape the entire tumor microenvironment. This perspective also suggests that biomarkers for establishing epigenetic drug effectiveness cannot be limited to cancer cell direct effects but must encompass quantifiable changes in the TME, including stromal composition or immune cell function.

## 7 Future directions in pancreatic cancer epigenetics

While the journey toward successfully utilizing epigenetic data for pancreatic cancer treatment goes on and is fraught with disappointments, the horizon beckons with some possible future directions. These fields endeavor to expand our understanding, improve therapeutic avenues, and eventually customize treatment to the patient.

### 7.1 Novel epigenetic targets and therapeutic modalities

The current toolbox of epigenetic drugs targets primarily established enzymes like DNMTs, HDACs, EZH2, and BET proteins. The epigenetic machinery is vast and complex and offers rich soil for finding new targets for therapy.

#### 7.1.1 Less-well-defined modifications and enzymes

Future studies should focus on enzymes involved in other histone modifications (e.g., specific writers, erasers, or readers of ubiquitination, phosphorylation, or less common methylations) or subunits of various chromatin remodeling complexes that are uncovered to be deranged in PDAC.

#### 7.1.2 Epitranscriptomics/RNA epigenetics

The field of RNA epigenetics, i.e., modifications of RNA molecules themselves (e.g., N6-methyladenosine, m6A), is expanding very rapidly. The enzymes that add, erase, or read these RNA modifications (e.g., METTL3, FTO, YTHDF proteins for m6A) are emerging as important regulators of gene expression and tend to get dysregulated in cancer. Inhibitors or modulators of such epitranscriptomic processes could afford new therapeutic prospects (Lan et al., 2019; 2021; Nombela et al., 2021).

#### 7.1.3 Non-coding RNAs as targets

While some ncRNAs are being explored as biomarkers, the modulation of dysregulated oncogenic lncRNAs or miRNAs (e.g., by antisense oligonucleotides, small interfering RNAs, or small molecules against their function or biogenesis) or reconstitution of tumor-suppressive ncRNAs represents another therapeutic prospect.

#### 7.1.4 Improved drug design

One of the key goals is to develop next-generation epigenetic drugs with increased selectivity for specific enzyme isoforms or complexes, improved pharmacological properties (e.g., improved bioavailability, longer half-life), and decreased off-target activity in order to limit toxicity.

#### 7.1.5 Novel delivery systems and modalities

Novel delivery systems, such as nanoparticle-based carriers, are under investigation to improve the targeted delivery of epigenetic drugs into cancer cells and maximize their therapeutic ratio. New therapeutic modalities like Proteolysis Targeting Chimeras (PROTACs) are under development as well. PROTACs are bi-functional molecules that induce the targeted degradation of a specific protein (e.g., epigenetic enzymes) by the ubiquitin-proteasome system, offering a unique mechanism of action in contrast to traditional enzyme inhibitors (Tomaselli et al., 2020; Vogelmann et al., 2020; Kabir et al., 2023).

### 7.2 Epigenome editing with CRISPR-dCas9 and related platforms

The advancement of epigenome editing tools, particularly the CRISPR-Cas9-based tools, has opened new avenues for precisely editing epigenetic marks within targeted genomic sites, offering a new platform for therapeutic intervention. In this approach, a catalytically inactive or “dead” Cas9 (dCas9) enzyme that can still bind to the DNA but not cut it when guided by a particular guide RNA (gRNA) is combined with an epigenetic effector domain. This effector domain could be an enzyme that writes, erases, or modifies epigenetic marks, such as a DNA methyltransferase or demethylase, histone acetyltransferase or deacetylase (e.g., HDAC1), or a histone methyltransferase or demethylase. By designing the gRNA to target a specific gene promoter or regulatory element, the dCas9-effector fusion protein can be precisely delivered to that location to reprogram the local epigenome and thus control the expression of the target gene. A proof-of-concept experiment has demonstrated the use of a dCas9-HDAC1 fusion protein to epigenetically downregulate the mutant KRAS promoter in cancer cells, leading to reduced KRAS expression and inhibition of cancer cell proliferation (Liu et al., 2021). Though epigenome editing is now largely in the preclinical stages of development, particularly for *in vivo*, this is a very powerful research tool and potentially game-changing therapeutic modality for the future. There are considerable hurdles to be overcome, including the provision of *in vivo* delivery of the editing machinery into tumor cells with high efficiency and safety, minimizing off-target binding and undesirable epigenetic modification at other parts of the genome, and knowing the long-term effects of such an intervention.

### 7.3 Personalization of epigenetic therapy

The ultimate goal of leveraging our understanding of epigenetics in pancreatic cancer is to move towards a more personalized or precision medicine approach. The significant inter-tumoral heterogeneity in PDAC underscores the need for treatments tailored to the specific molecular characteristics of an individual patient’s tumor. Epigenetics plays a key role in this vision:

#### 7.3.1 Patient stratification

Comprehensive epigenetic profiling of patient tumors (e.g., using DNA methylation arrays, histone modification mapping, or ncRNA expression analysis) can help to stratify patients into distinct molecular subtypes. These subtypes may exhibit differential sensitivities to specific epigenetic drugs or combination therapies, allowing for more targeted treatment selection (Bailey et al., 2016; Jamali et al., 2024).

#### 7.3.2 Biomarker-guided therapy

Identifying predictive epigenetic biomarkers that can forecast a patient’s response to a particular epigenetic therapy is crucial. This would enable clinicians to select the most appropriate treatment based on the dominant epigenetic vulnerabilities identified in their tumor (Andricovich et al., 2018).

#### 7.3.3 Dynamic monitoring

Liquid biopsies that detect circulating epigenetic markers (e.g., cfDNA methylation patterns or circulating ncRNAs) could be used to dynamically monitor a patient’s response to epigenetic therapy in real-time, detect the emergence of resistance mechanisms early, and allow for timely adjustments to the treatment plan (Shen et al., 2018).

#### 7.3.4 Integration of multi-omics data

Realizing the full potential of personalized epigenetic therapy will require the integration of multi-omics data, including genomics (mutational status), epigenomics, transcriptomics (gene expression), and proteomics, with advanced bioinformatics tools and artificial intelligence (AI) algorithms. This will enable a more comprehensive understanding of each patient’s tumor biology and facilitate the development of predictive models for treatment response (Hasin et al., 2017; Maher et al., 2025).

The interplay of the epigenome with cellular metabolism and the host microbiome represents rapidly advancing frontiers in cancer research that will undoubtedly intersect with pancreatic cancer epigenetics. Epigenetic enzymes rely on metabolic intermediates as essential cofactors (e.g., S-adenosylmethionine (SAM) for methylation reactions, acetyl-CoA for acetylation, α-ketoglutarate for certain demethylases) (Reid et al., 2017). Cancer cells are known to undergo significant metabolic reprogramming to fuel their growth and survival (Lyssiotis and Kimmelman, 2017). These altered metabolic states can directly influence the availability of these cofactors, thereby impacting the epigenetic landscape and gene expression programs. Understanding and potentially targeting these metabolic-epigenetic linkages could offer novel therapeutic strategies (Halbrook and Lyssiotis, 2017). Similarly, the gut and tumor microbiome are increasingly recognized for their ability to influence host epigenetics, immune responses, and even the efficacy of cancer therapies (Zheng et al., 2020; Attebury and Daley, 2023). Future research in pancreatic cancer will likely need to integrate these additional layers of complexity, the metabolome and the microbiome, for a more holistic understanding of the disease and to uncover new vulnerabilities or modulatory approaches that could complement epigenetic therapies.

## 8 Discussion

The journey into the rich epigenetic universe of pancreatic cancer has uncovered a dynamic, complex but potentially tractable universe of regulatory mechanisms that are inextricably intertwined with the aggressive behavior and lethal course of cancer. From the aberrations in DNA methylation and the miswriting of the histone code in the global scale to the misregulation of a vast network of non-coding RNAs, all the epigenetic alterations are not bystanders but rather players that actively engage in tumorigenesis, intra-tumoral heterogeneity, metastatic dissemination, and orchestration of immune evasion.

Although difficulties in successfully treating pancreatic cancer continue to be daunting, the built-in reversibility of epigenetic marks provides a novel therapeutic opportunity, in contrast to the frequently permanent nature of genetic mutations. Continued progress and optimization of epigenetic biomarkers, especially those suitable for detection by liquid biopsies, are very promising for facilitating earlier diagnosis, enhancing prognostication accuracy, and directing patient stratification for precise therapies.

Furthermore, a growing armamentarium of epigenetic drugs, acting on more than one component of the epigenetic machine, are beginning to show promise. While effectiveness with single-agent therapy has been modest in this disease with refractoriness, the ultimate value of these agents in all likelihood lies in their use in combination with standard treatment approaches like chemotherapy and novel approaches like immunotherapy. These combinations aim to resensitize tumors to cytotoxins, remodel the tumor immunosuppressive milieu, and overcome the universal challenge of therapeutic resistance.

The path forward is illuminated by exciting directions to come. The discovery of fresh epigenetic targets, the advent of precision epigenome editing tools like CRISPR-dCas9, and the long-term goal of bringing in-depth epigenetic knowledge into truly personalized medicine approaches all provide a sense of cautious optimism. While the biological complexity of pancreatic cancer is immense and the clinical obstacles are formidable, concerted, persistent effort in researching its epigenetic roots is a necessity. It is only through concerted efforts that scientific advancements can be translated into useful clinical dividends, ultimately a step towards achieving some change from the grim prognosis for patients fighting this formidable malignancy.

## Glossary

5mC: 5-methylcytosine
AI: Artificial Intelligence
BET: Bromodomain and Extra-Terminal Motif
CAF: Cancer-Associated Fibroblast
cfDNA: Cell-Free DNA
CSC: Cancer Stem Cell
DNMT: DNA Methyltransferase
DNMTi: DNA Methyltransferase Inhibitor
ECM: Extracellular Matrix
EUS-FNA: Endoscopic Ultrasound–Guided Fine-Needle Aspiration
EMT: Epithelial-to-Mesenchymal Transition
EZH2: Enhancer of Zeste Homolog 2
EZH2i: EZH2 Inhibitor
FDA: U.S. Food and Drug Administration
HAT: Histone Acetyltransferase
HDAC: Histone Deacetylase
HDACi: Histone Deacetylase Inhibitor
HMT: Histone Methyltransferase
HMTi: Histone Methyltransferase Inhibitor
ICIs: Immune Checkpoint Inhibitors
IPMA: Intraductal Papillary Mucinous Adenoma
IPMC: Intraductal Papillary Mucinous Carcinoma
IPMN: Intraductal Papillary Mucinous Neoplasm
lncRNA: Long Non-Coding RNA
m6A: N6-Methyladenosine
MBD: Methyl-CpG-Binding Domain
MDM: Methylated DNA Marker
miRNA: MicroRNA
ncRNA: Non-Coding RNA
PanIN: Pancreatic Intraepithelial Neoplasia
PDAC: Pancreatic Ductal Adenocarcinoma
PD-L1: Programmed Death-Ligand 1
PNET: Pancreatic Neuroendocrine Tumor
PRC2: Polycomb Repressive Complex 2
PRMT: Protein Arginine Methyltransferase
PROTAC: Proteolysis Targeting Chimera
SAM: S-Adenosylmethionine
TGF-β: Transforming Growth Factor Beta
TME: Tumor Microenvironment
TSG: Tumor Suppressor Gene
UTR: Untranslated Region
